# Impact of Mango Puree Supplementation on Inflammatory, Muscle Damage, and Selected T-Cell Biomarkers in Elite Beach Volleyball Players During Regular Training

**DOI:** 10.3390/nu18030525

**Published:** 2026-02-04

**Authors:** Wason Parklak, Saksayam Sawaengwaisayasuk, Nattapong Chaipatpreecha, Bandhita Wanikorn, Surat Komindr, Narongsuk Munkong, Watunyou Khamros, Teeraphan Sangkaew, Metawee Duangjinda, Surasawadee Somnuk

**Affiliations:** 1 Research Center for Non-Infectious Diseases and Environmental Health, Research Institute for Health Sciences, Chiang Mai University, Chiang Mai 50200, Thailand; wason.p@cmu.ac.th; 2 Department of Sports Science, Sports Authority of Thailand, Bangkok 10240, Thailand; saksayam@sat.or.th (S.S.); nattapong.c@sat.or.th (N.C.); 3 Department of Biotechnology, Faculty of Agro-Industry, Kasetsart University, Bangkok 10900, Thailand; agibtw@ku.ac.th; 4 Division of Nutrition and Biochemical Medicine, Department of Medicine, Faculty of Medicine, Ramathibodi Hospital, Mahidol University, Bangkok 10400, Thailand; surat.kom@mahidol.ac.th; 5 Department of Pathology, School of Medicine, University of Phayao, Phayao 56000, Thailand; narongsuk.mu@up.ac.th; 6 Department of Sports Science, Faculty of Physical Education, Sports and Health, Srinakharinwirot University, Nakhon Nayok 26120, Thailand; watunyou@g.swu.ac.th; 7 Faculty of Education, Thaksin University, Songkhla 90000, Thailand; teeraphan@tsu.ac.th; 8 Faculty of Nursing, Kanjanaburi Western University, Huai Kra Jao, Kanchanaburi 71170, Thailand; metawee.du@western.ac.th; 9 Department of Sports Science, Faculty of Sports and Health Science, Kasetsart University, Kamphaeng Saen Campus, Nakhon Pathom 73140, Thailand

**Keywords:** mango, Nam Dok Mai, inflammatory markers, muscle damage, immune response, selected T-cell biomarkers, beach volleyball players, athletes

## Abstract

**Background/Objectives**: Mango is a tropical fruit rich in polyphenols and carotenoids that may support recovery-related physiological responses during athletic training. This study examined the effects of mango puree supplementation on inflammatory biomarkers, muscle damage, and selected T-cell subsets in Thai men’s national beach volleyball players during regular training. **Methods**: Fifteen male athletes completed a pilot randomized, single-blind, crossover trial. Participants consumed the mango puree or placebo (600 g/day) for 4 weeks, separated by a 2-week washout period. Blood samples and physiological measurements were collected at baseline and at the end of each intervention period. Outcomes were analyzed using linear mixed-effects models. **Results**: Mango puree supplementation was associated with lower concentrations of C-reactive protein (mean difference: −1.6 mg/L; 95% CI: −2.1 to −1.1; *p* < 0.001), interleukin-6 (−0.7 pg/mL; 95% CI: −1.2 to −0.3; *p* = 0.003), and creatine kinase (−290.1 U/L; 95% CI: −356.1 to −224.1; *p* < 0.001) compared with the placebo. The percentage of CD4+ T cells (9.82 percentage points; 95% CI: 5.0 to 14.6; *p* < 0.001) and the CD4/CD8 ratio (0.37; 95% CI: 0.11 to 0.63; *p* = 0.007) were higher during mango puree supplementation, while CD8+ T-cell percentage did not differ between conditions. No significant treatment effects were observed for body composition parameters or blood pressure (all *p* > 0.05). Total energy intake remained unchanged across intervention periods (*p* > 0.05). **Conclusions**: Mango puree supplementation during regular training was associated with lower inflammatory and muscle damage biomarkers and alterations in selected T-cell subsets compared with the placebo.

## 1. Introduction

Nutrition plays a vital role in promoting both general health and athletic performance. Beach volleyball, an outdoor sport played on sand, is physically demanding and relies heavily on both anaerobic and aerobic energy systems. It requires explosive strength, muscular endurance, agility, and sustained muscle activation. These demands place athletes—particularly national-level players—at an elevated risk of muscle damage, inflammation, and immune alterations due to repeated eccentric contractions, mechanical stress, and environmental exposure [1,2]. Exercise-induced stress is associated with increased production of reactive oxygen species (ROS), which, when not adequately neutralized by antioxidants, can lead to oxidative stress. This condition disrupts cellular integrity and triggers inflammation through damage to proteins, lipids, and nucleic acids [3]. Excessive oxidative stress impairs muscle contraction, induces fatigue, and diminishes performance [4].

One of the early signs of muscle damage is elevated levels of creatine kinase (CK), which typically increases following intense training [5]. The inflammatory response also involves the release of cytokines, especially interleukin-6 (IL-6), produced by skeletal muscles and immune cells, serving both pro- and anti-inflammatory roles [6]. Importantly, IL-6 exhibits complex, context-dependent kinetics: it is acutely released from contracting skeletal muscle as a myokine with metabolic and anti-inflammatory functions during exercise, while chronically elevated resting IL-6 concentrations are more closely associated with systemic inflammation and inadequate recovery [7]. Additionally, strenuous exercise can stimulate hepatic production of C-reactive protein (CRP), a systemic marker of acute inflammation [8]. If this inflammatory response is prolonged, particularly with continuous T cell activation, it may disrupt metabolic regulation and lead to muscle atrophy and impaired recovery [9]. To mitigate these effects, athletes require not only adequate caloric intake but also specific micronutrients and phytochemicals that support immune function and reduce oxidative damage. Antioxidant-rich foods, particularly fruits containing polyphenols, carotenoids, and vitamins C and E, may help reduce exercise-induced inflammation and promote recovery [10]. Fruit-based interventions rich in polyphenols (e.g., tart cherry, blueberry) have been investigated for their potential to attenuate exercise-induced muscle damage, oxidative stress, and inflammation, particularly during periods of intensified training [11,12,13].

Mango (*Mangifera indica*) is a tropical fruit widely appreciated for its sensory appeal and nutritional value. It contains carbohydrates, fiber, vitamins (B, C, and E), minerals, and polyphenolic compounds with potent antioxidant properties [14]. Studies suggest that polyphenols in mango may reduce CRP levels and suppress inflammation [15]. Among mango varieties, the Nam Dok Mai cultivar is particularly popular in Thailand. It is consumed ripe and contains high levels of β-carotene and polyphenols, contributing to its antioxidant and anti-inflammatory potential [16,17]. A typical serving (~90 g with skin or ~79 g edible portion) provides essential nutrients and bioactives that support immune defense and muscle function. Vitamins C and E in mango act synergistically to protect cardiac and skeletal muscles from oxidative damage [18,19], while β-carotene enhances mitochondrial energy metabolism [20]. Furthermore, mango flavonoids may improve glycemic control and microcirculatory function, which are relevant to athletes undergoing intense physical exertion [21,22].

Despite growing evidence supporting the use of polyphenol-rich fruits in sports nutrition, data specifically examining mango supplementation in elite athletes remain scarce. Moreover, no studies to date have evaluated fruit-based interventions in elite beach volleyball players during an actual training camp, where cumulative mechanical load, environmental exposure, and limited recovery time may amplify chronic inflammatory stress. The optimal biomarkers and intervention duration to capture these training-related adaptations also remain uncertain. CK, IL-6, and CRP are commonly used to reflect cumulative muscle damage and systemic inflammatory status under sustained training loads [23]. A 4-week intervention period is commonly used in nutritional studies to evaluate cumulative training-related changes in inflammatory and muscle damage markers under stable training conditions [24,25]. Therefore, this study aimed to investigate the effects of 4-week mango puree supplementation on biomarkers of muscle damage (CK), inflammation (IL-6 and CRP), and immune-related parameters in Thai national male beach volleyball players undergoing regular intensive training. By addressing a clearly defined population, real-world training context, and biologically relevant resting biomarkers, this study seeks to fill an important gap in the evidence supporting fruit-based nutritional strategies for elite athletes.

## 2. Materials and Methods

### 2.1. Preparation of Mango Puree

Ripe Nam Dok Mai mangoes at ripening stage 3, characterized by uniform maturity, firm texture, balanced sweet–sour flavor, and free from bruises or spoilage, were procured from a local fresh market in Bangkok, Thailand. The mangoes were thoroughly washed with tap water to remove any surface contaminants, peeled, and deseeded. The edible pulp was collected and blended using a high-speed blender until a smooth, homogeneous puree was obtained. The puree was then passed through a fine mesh sieve to ensure uniform texture. No sugar, water, preservatives, or additives were incorporated during the preparation process. The prepared mango puree was portioned into sterilized food-grade containers at 200 g per serving, sealed, and stored at –20 °C until further use for nutritional analysis and subsequent consumption by study participants.

Samples were randomly collected from three independent preparation batches of prepared mango puree for analysis. The bioactive constituents and nutritional value of the mango puree were determined at the Central Laboratory, Faculty of Agriculture, Kasetsart University, Bangkok, Thailand. The results were expressed as mean ± standard deviation (SD).

### 2.2. Ethical Approval

This clinical study was conducted in accordance with the principles outlined in the Declaration of Helsinki and was approved by the Kasetsart University Research Ethics Committee, Kasetsart University (IRB approval number: COA64/041; approval date: 1 July 2021). All participants were fully informed about the study’s objectives, procedures, potential benefits, and risks. Participation was entirely voluntary, and individuals were free to withdraw at any time without consequence. Informed consent was voluntarily obtained from all participants prior to the study.

### 2.3. Study Subjects

A total of 15 male Thai national beach volleyball players were recruited to participate in this pilot study. While the sample size is limited, it is comparable to previous exploratory studies examining the effects of dietary and antioxidant supplementation on exercise-induced oxidative stress and related outcomes in athletic populations [26,27]. Participants were eligible for inclusion if they were between 18 and 30 years of age, actively training as members of the Thai national beach volleyball team during the designated training camp period, and in good general health with no known chronic medical conditions. Participants were required to be free from any injuries that could interfere with study participation and to provide written informed consent, indicating their willingness to take part in the study. Individuals were excluded if they had any illness or medical condition that might influence the study outcomes or if they experienced gastrointestinal disorders such as chronic constipation, diarrhea, inflammatory bowel disease, or other persistent digestive issues. Discontinuation criteria included the development of adverse effects related to mango puree consumption—such as gastrointestinal discomfort or intolerance—or failure to adhere to the study protocol.

### 2.4. Study Design

This pilot study employed a randomized, single-blind, crossover design. Participants were blinded to the intervention sequence, while investigators were aware of group allocation. Prior to the intervention, participants completed a dietary assessment using a 24-h food recall questionnaire. Body composition was measured using a bioelectrical impedance analyzer (InBody 720, Biospace Co., Ltd., Pleasanton, CA, USA). Baseline blood samples were collected to assess inflammatory markers, muscle damage biomarkers, and T cell subsets, specifically CD4+ and CD8+ lymphocyte counts. All blood analyses were performed at National Healthcare Systems Co., Ltd., Bangkok, Thailand. All participants received both the intervention and control conditions in two separate periods, with a two-week washout interval between them to minimize potential carryover effects. The two-week washout period was selected based on previous crossover studies and was intended to minimize potential carryover effects. Potential period and sequence effects were further addressed in the statistical analysis.

The intervention consisted of ripe Nam Dok Mai mango puree, while the control (placebo) was a fruit-flavored beverage matched for volume and approximate energy content. The placebo beverage was prepared using water, maltodextrin, citric acid, food-grade thickening agents, and mango-orange flavoring. Food-grade thickening agents were added to adjust viscosity to a level comparable to that of the mango puree. The energy content of the placebo was calculated using the INMUCAL–Nutrients Version 4.0 database to approximate the caloric content of the mango puree. Both treatments were administered at a total daily dose of 600 g, divided into three servings of 200 g each. Participants were instructed to consume each serving at fixed time points: one hour before morning training, during the morning training session, and one hour before evening training. On non-training days (Saturday afternoon and Sunday), participants continued to consume the assigned supplementation at three standardized time points corresponding to the usual training-day schedule to ensure consistency of daily intake throughout the intervention period. Supplement compliance was monitored through daily supervision by team staff and by collecting returned packaging at the end of each intervention period. No participant discontinued the intervention, and all participants returned empty containers, indicating full consumption of the provided portions during both intervention phases. Throughout the study, participants were advised to maintain a consistent dietary pattern aside from the assigned intervention to minimize dietary confounding variables.

**Figure 1:**
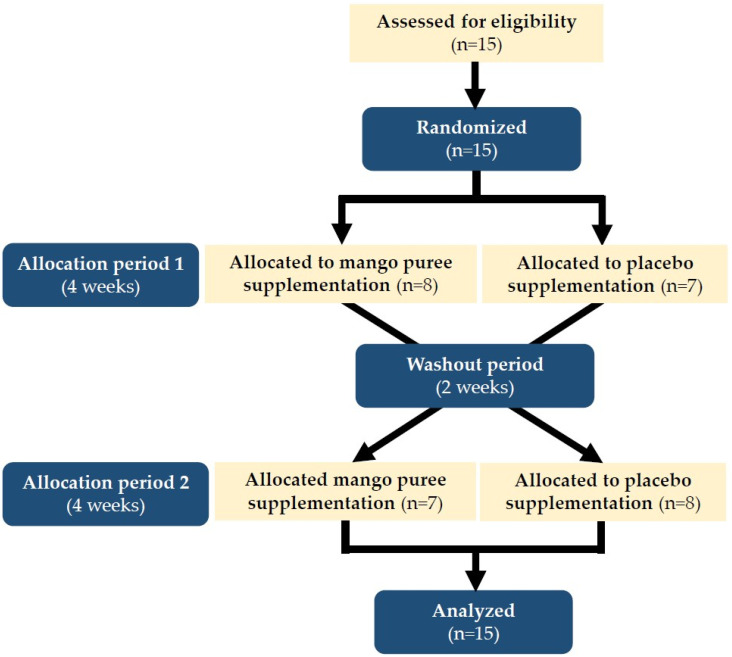
Participant flow diagram for the randomized, single-blind, crossover trial of mango puree and placebo supplementation.

Participants were randomly assigned to one of two groups using a computer-generated randomization sequence. During the first 4-week period, Group 1 received the mango puree along with 150 mL of plain water per serving, while Group 2 received the placebo control beverage with 150 mL of plain water. At the end of the first 4-week period, dietary intake, physiological measurements, and blood samples were reassessed to evaluate inflammatory markers, muscle damage biomarkers, and CD4+ and CD8+ T cell levels, using procedures identical to those performed at baseline. The two-week washout period was selected based on previous crossover studies in athletes and healthy adults investigating nutritional or fruit-based interventions, which reported sufficient time for biomarker stabilization and minimization of carryover effects [28,29]. After the two-week washout period, participants crossed over to the alternate treatment for another 4-week intervention period. Upon completion of the second intervention period, final assessments were conducted, including dietary intake, physiological measurements, and blood sample collection, using the same procedures as those performed at baseline and at the end of the first intervention period. All physiological measurements and blood samples were collected in the morning under resting conditions, prior to training sessions, to minimize acute exercise-related variability. The overall randomized, single-blind, crossover design is illustrated in Figure 1.

The male Thai national beach volleyball players who participated in this study were engaged in a regular national team training program. All participants had been following this standardized training program for at least one month prior to study enrollment, ensuring training familiarity and stabilization before the intervention. The athletes trained twice daily—morning and evening sessions—for approximately 3 h per session, 5.5 days per week, from Monday to Saturday morning.

The structured training schedule was as follows:

- Weight training was conducted on Tuesday and Thursday mornings.
- Physical conditioning sessions took place every morning from Monday to Saturday.
- Skill-based volleyball training was held during the evening sessions.

Athletes had rest periods on Saturday afternoons and the entire day on Sundays, during which no training sessions were scheduled.

### 2.5. Assessment of Dietary Intake

Dietary intake data were collected using three consecutive 24-h dietary recalls, covering two weekdays and one weekend day to capture variations in eating patterns. Prior to the assessment, participants were instructed to record or photograph all foods and beverages consumed for familiarization purposes for at least one week to enhance recall accuracy and minimize memory-related bias. During each recall session, trained research staff conducted face-to-face interviews using standardized questionnaires and portion size estimation tools, including food models, measuring cups, plates, and spoons. Dietary intake was assessed at three time points: prior to the intervention (baseline), at the end of Supplementation Period 1, and at the end of Supplementation Period 2. Nutrient intake data were analyzed using INMUCAL–Nutrients Version 4.0, a validated dietary analysis software developed by the Institute of Nutrition, Mahidol University, Thailand. The software provided estimates of energy and nutrient intake, and results were expressed as average daily intake per participant.

### 2.6. Statistical Analysis

All statistical analyses were performed using SPSS for Windows, version 21.0 (IBM Corp., Armonk, NY, USA). Primary outcome variables, including inflammatory, muscle damage, immune, and physiological parameters, were analyzed using linear mixed-effects models, with participant included as a random intercept and treatment, period, and sequence specified as fixed effects; baseline values were included as covariates. Results from these models are presented as estimated marginal means and adjusted mean differences with 95% confidence intervals. Dietary intake variables were analyzed using paired *t*-tests to assess differences between baseline and each intervention period and to compare mango puree and placebo conditions. These analyses were performed to confirm the absence of systematic dietary changes across study periods.

## 3. Results

### 3.1. Bioactive Compounds and Nutritional Value of the Mango Puree

The bioactive compounds and nutritional composition of the mango puree and placebo used in the intervention are presented in Table 1. The mango puree provided 167.86 ± 9.71 kcal per 200 g serving, with carbohydrates accounting for 39.68 ± 2.52 g, along with 0.42 ± 0.01 g of fat and 1.34 ± 0.10 g of protein (%N × 6.25). The moisture content was 157.76 ± 4.56 g, and the mango puree contained 33.06 ± 4.08 g of total sugars and 0.71 ± 0.02% starch. Potassium, β-carotene, and total phenolic compounds were measured at 254.00 ± 17.35 mg, 746.00 ± 37.16 µg, and 104.72 ± 9.49 mg, respectively. The placebo provided 166.80 kcal per 200 g serving and 41.70 g of carbohydrates, with no fat or protein. The moisture content was 158.60 g. Potassium content was <10 mg, and starch, β-carotene, and total phenolic compounds were not determined.

### 3.2. General Characteristics and Body Composition Parameters

Table 2 summarizes the adjusted general characteristics and body composition parameters following mango puree and placebo supplementation, as estimated by linear mixed-effects models. The participants were young adult male national beach volleyball players, with a mean age of 24 ± 3 years and an average of 4 ± 3 years of experience in the national team. No significant treatment effects were observed for body weight, waist–hip ratios, muscle mass, fat mass, percent body fat, protein mass, mineral content, total body water percentage, systolic blood pressure, or diastolic blood pressure (all *p* > 0.05). In contrast, heart rate was significantly higher during the mango puree condition compared with the placebo condition (mean difference: 3 bpm; 95% CI: 0 to 7; *p* = 0.042).

### 3.3. Energy and Nutrient Intake

In Table 3, the distribution of energy-yielding macronutrients did not differ significantly among the three study periods (*p* > 0.05). During the baseline period, the proportions of energy derived from carbohydrates, fat, and protein were 42.9 ± 12.8%, 31.1 ± 10.9%, and 26.0 ± 10.3%, respectively. During the mango puree period, the corresponding values were 46.1 ± 11.9% for carbohydrates, 24.8 ± 5.2% for fat, and 29.1 ± 12.8% for protein. During the placebo period, carbohydrate, fat, and protein contributions were 39.7 ± 13.6%, 37.4 ± 14.7%, and 22.9 ± 7.9%.

Total energy intake and absolute macronutrient intake during each study period are shown in Table 4. Mean energy intake was 2066 ± 609 kcal during the baseline period, 2074 ± 630 kcal during the mango puree period, and 2058 ± 588 kcal during the placebo period. No statistically significant differences were observed in total energy intake or in carbohydrate, fat, and protein intake among the study periods (*p* > 0.05).

### 3.4. Effects of Mango Puree Supplementation on Inflammatory and Muscle Damage Biomarkers

**Figure 2:**
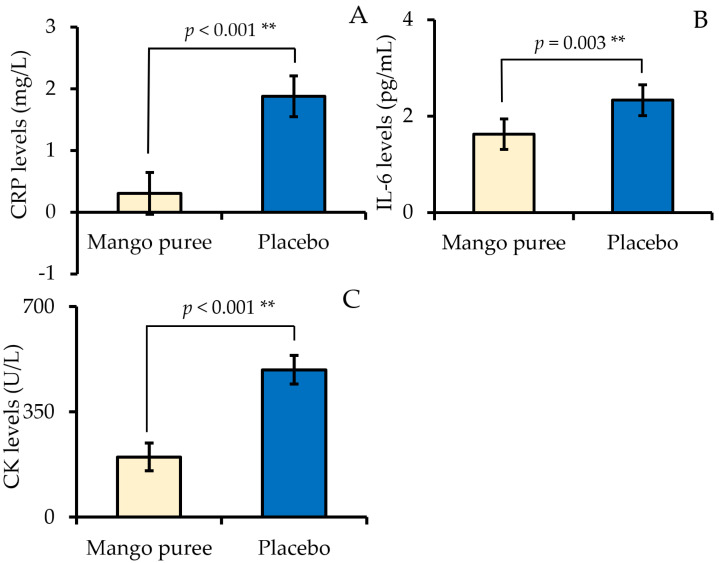
Adjusted concentrations of C-reactive protein (CRP) (**A**), interleukin-6 (IL-6) (**B**), and creatine kinase (CK) (**C**) following mango puree and placebo supplementation. Data are presented as adjusted means with 95% CI derived from linear mixed-effects models. *p* values refer to the fixed effect of treatment. ** indicates *p* < 0.01.

Figure 2 presents the adjusted concentrations of CRP, IL-6, and CK following mango puree and placebo supplementation, as estimated by linear mixed-effects models. CRP concentrations were significantly lower in the mango puree condition compared with placebo, with a mean difference of −1.6 mg/L (95% CI: −2.1 to −1.1, *p* < 0.001). Similarly, IL-6 levels were lower after mango puree supplementation compared with the placebo, with a mean difference of −0.7 pg/mL (95% CI: −1.2 to −0.3, *p* = 0.003). CK concentrations were also reduced in the mango puree condition relative to the placebo, with a mean difference of −290.1 U/L (95% CI: −356.1 to −224.1, *p* < 0.001).

### 3.5. Effects of Mango Puree Supplementation on Selected T-Cell Subsets

**Figure 3:**
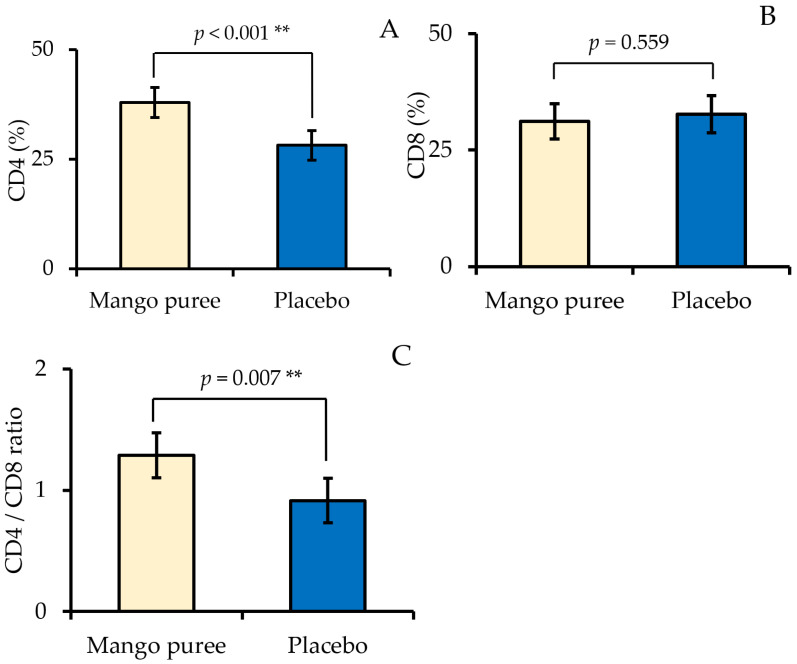
Adjusted percentages of CD4 (**A**), CD8 (**B**), and the CD4/CD8 ratio (**C**) following mango puree and placebo supplementation. Data are presented as adjusted means with 95% confidence intervals derived from linear mixed-effects models. *p* values refer to the fixed effect of treatment. ** indicates *p* < 0.01.

Figure 3 presents the adjusted percentages of CD4+ and CD8+ T cells and the CD4/CD8 ratio following mango puree and placebo supplementation, as estimated by linear mixed-effects models. The percentage of CD4+ T cells was significantly higher during the mango puree condition compared with the placebo condition, with a mean difference of 9.82 percentage points (95% CI: 5.0 to 14.6; *p* < 0.001). No significant difference was observed in the CD8+ T-cell percentage between mango puree and placebo supplementation (mean difference: −1.6 percentage points; 95% CI: −7.1 to 4.0; *p* = 0.559). The CD4/CD8 ratio was significantly greater during mango puree supplementation compared with the placebo, with a mean difference of 0.37 (95% CI: 0.11 to 0.63; *p* = 0.007).

## 4. Discussion

Mango is a tropical fruit widely recognized for its rich nutritional profile, providing essential vitamins, minerals, and dietary fiber [10]. In addition to these nutrients, mango is a notable source of bioactive compounds, particularly phenolic compounds and β-carotene, which have been extensively reported to exhibit antioxidant and anti-inflammatory properties [30,31]. Nam Dok Mai mango, a popular cultivar commonly consumed in Thailand and Southeast Asia, has been shown to contain substantial levels of carotenoids and phenolic constituents, contributing to its strong antioxidant capacity [16,17]. Previous research has demonstrated that mango-derived phenolics and carotenoids may modulate oxidative stress and inflammatory responses, which are key physiological challenges during periods of intensive physical training, particularly in athletes exposed to repeated high training loads [18,20,32].

Athletes engaged in high-intensity and structured training programs, such as elite beach volleyball players, are frequently exposed to repetitive mechanical loading, muscle microdamage, and exercise-induced inflammation, particularly during pre-competition or training camp phases [33,34]. These physiological stresses are often associated with elevations in inflammatory and muscle damage biomarkers and may compromise recovery if not adequately managed [5,6,8]. Therefore, dietary strategies incorporating fruits rich in antioxidant and anti-inflammatory compounds have been proposed as a supportive approach to help attenuate training-related physiological stress in this population.

In the present study, mango puree was consumed continuously for 4 weeks, and all participants completed the prescribed intake of three servings per day, resulting in full compliance with the intervention protocol. No adverse effects, including gastrointestinal discomfort or other treatment-related symptoms, were reported throughout the study period. Each 200 g serving of mango puree provided 746.0 ± 37.2 µg of β-carotene and 104.7 ± 9.5 mg of total phenolic compounds. Based on the prescribed intake of three servings per day, the corresponding daily intakes were 2242.0 ± 111.1 µg of β-carotene and 323.6 ± 16.6 mg of total phenolic compounds. Previous studies have reported that β-carotene and phenolic compounds from fruits and vegetables are associated with reductions in oxidative stress and inflammatory biomarkers [35,36], as well as improvements in recovery following strenuous exercise [37]. These findings provide a plausible nutritional basis through which mango puree supplementation may have contributed to the modulation of inflammatory, muscle damage, and immune-related biomarkers observed in athletes undergoing intensive training.

In the present study, mango puree supplementation did not result in statistically significant changes in body composition parameters, including body weight, waist–hip ratio, muscle mass, fat mass, percent body fat, protein mass, or mineral content, compared with baseline values. These findings differ from previous reports demonstrating changes in body weight or adiposity following mango or mango-derived product supplementation in animal models and non-athletic human populations [38,39,40]. The lack of observable changes in body composition may be explained by differences in study population and physiological context. The participants were elite athletes with low baseline fat mass, high habitual energy expenditure, and sustained training loads, conditions under which meaningful alterations in body composition are less likely to occur over short-to-moderate time frames. In addition, the intervention was conducted during regular training, during which training-related adaptations and high energy turnover may attenuate or mask subtle dietary effects on body composition [41,42]. Importantly, structural outcomes such as body composition generally require prolonged or cumulative stimuli to become detectable, whereas circulating biomarkers reflect more dynamic physiological responses. Previous nutrition and exercise studies have shown that inflammatory and muscle damage biomarkers can respond within weeks of dietary intervention, even in the absence of concurrent changes in body composition [43,44]. This distinction may account for the significant effects observed on inflammatory and immune-related biomarkers in the present study, despite the absence of changes in body composition.

Resting heart rate is widely used as an indicator of autonomic balance and training status in athletes. Measures of resting heart rate and related autonomic activity are sensitive to variations in training load and recovery, and are commonly applied in monitoring fatigue and adaptation during athletic training cycles [45]. Additionally, cardiac parasympathetic reactivation following exercise has been shown to vary with fitness and training status, indicating that heart rate responses reflect recovery dynamics rather than static cardiovascular changes [46]. Therefore, the modest increase in resting heart rate observed during the mango puree condition may reflect normal physiological variability associated with training load and recovery status rather than a direct effect of the intervention.

Although SBP did not differ significantly between conditions, a modest trend toward higher SBP was observed during the mango puree period. Given that training load and total energy intake were comparable between periods and that the placebo provided slightly higher sugar per serving, this trend is unlikely to be explained by differences in training stimulus or caloric intake. In normotensive, highly trained athletes, resting SBP is subject to short-term physiological variability and is sensitive to measurement standardization [47]. Psychological stress and sleep characteristics, which are known to influence blood pressure regulation, were not assessed in the present study and may have contributed to the observed variability [48,49]; these factors warrant consideration in future research. Importantly, SBP values remained within the normotensive range, and no concurrent changes were observed in diastolic blood pressure or body composition, suggesting that the observed SBP trend reflects physiological variability rather than a clinically meaningful blood pressure effect.

Exercise-induced muscle damage and low-grade inflammation are common responses to sustained high training loads, with biomarkers such as CRP and IL-6 commonly used to reflect systemic inflammatory status in athletes [6,8,33,34]. In the present study, mango puree supplementation was associated with lower adjusted concentrations of CRP and IL-6 compared with placebo, as estimated by linear mixed-effects models. However, baseline CRP values were within the low or near-normal range, indicating the absence of overt systemic inflammation at study entry. Accordingly, these findings should be interpreted as modulation of resting inflammatory markers during the training period rather than evidence of a definitive anti-inflammatory effect. In addition, blood samples were collected at rest at the end of each intervention period, capturing chronic rather than acute post-exercise responses, which may be influenced by diurnal variation and the timing of the last training session. In volleyball athletes, biomarker responses should also be interpreted within a sport-specific physiological context. Recent evidence has highlighted that biological markers in volleyball players may reflect integrated responses to physical load, neuroendocrine regulation, and recovery status rather than isolated inflammatory processes, underscoring the complexity of biomarker interpretation in this population [50].

CK, a commonly used marker of muscle damage [5], was lower during the mango puree period compared with the placebo, while changes relative to baseline were modest. Similar patterns have been reported in studies of polyphenol-rich fruit interventions, particularly in well-trained athletes, where between-condition differences in CK are more apparent than within-condition changes from baseline [51,52]. Given the high training status of the participants, baseline CK levels may reflect chronic adaptation to repeated loading, limiting the magnitude of detectable changes. Thus, the lower CK concentrations observed relative to the placebo suggest a more favorable muscle damage profile during mango puree supplementation rather than complete attenuation of exercise-induced muscle damage. Overall, the findings indicate that mango puree supplementation was associated with differences in selected inflammatory and muscle damage biomarkers under regular training conditions. These results should be interpreted cautiously within the context of low baseline inflammation, training-related variability, and the exploratory nature of the study.

Evaluation of CD4+ and CD8+ T-cell percentages and the CD4/CD8 ratio is commonly used to describe aspects of adaptive immune balance in athletes, as alterations in these markers have been associated with training stress, recovery status, and susceptibility to infection [53,54]. In the present study, linear mixed-effects model analysis demonstrated that mango puree supplementation was associated with higher adjusted percentages of CD4+ T cells and a greater CD4/CD8 ratio compared with the placebo, whereas the CD8+ T-cell percentage did not differ between conditions. These findings indicate a modulation of selected adaptive immune cell distributions during mango puree supplementation under regular training conditions. However, given that only T-cell subsets were assessed and no markers of innate or functional immunity were measured, the physiological significance of these changes should be interpreted with caution. Similar variations in T-cell-related markers have been reported in trained athletes and may reflect transient adaptations to training load, nutritional factors, or recovery status rather than definitive changes in immune function [55,56]. Further studies incorporating a broader range of immune markers are needed to clarify the relevance of these observations.

Although dietary intake was not strictly controlled, the athletes participated in a national team training camp where ongoing nutritional education and guidance were provided by professional sports nutritionists. Dietary assessment using repeated 24-h food recalls indicated an average reported daily energy intake of approximately 2066 kcal, with no significant differences in total energy or macronutrient intake between baseline, mango puree, and placebo periods. Given the known limitations of 24-h dietary recalls in elite athletes, particularly during intensive training, these values should be interpreted as reported intake and may underestimate absolute energy consumption. Nevertheless, the consistency of dietary intake across study periods suggests that major changes in habitual energy or macronutrient intake were unlikely to confound the observed treatment effects.

The macronutrient distribution presented in Table 3 reflects a reported dietary pattern characterized by relatively lower carbohydrate intake and comparatively higher proportions of fat and protein across all study periods. While carbohydrate intake appeared lower than levels commonly recommended to optimize muscle glycogen availability, such patterns have been increasingly reported in elite athletes during intensive training phases and may be influenced by practical constraints such as training-induced fatigue, appetite suppression, and limited eating opportunities [2,57,58]. Importantly, the absence of differences in macronutrient distribution between intervention periods supports the interpretation that the observed differences in inflammatory and muscle damage biomarkers were unlikely to be driven by variations in habitual macronutrient intake, but rather occurred within a relatively stable dietary context.

This study has several limitations. First, the sample size was relatively small, as the study was conducted in a specific population of Thai men’s national beach volleyball players and included the entire eligible team (*n* = 15); therefore, the findings should be interpreted as exploratory. Future studies involving larger and more diverse athletic populations are warranted to confirm and extend these observations. Second, dietary intake was assessed using 24-h dietary recalls, which are subject to recall bias, particularly in elite athletes with high training loads. Food intake surrounding training sessions (pre-, during-, and post-exercise) may not have been fully captured, potentially leading to an underestimation of absolute energy intake, as reflected by the relatively low reported daily energy intake (~2000 kcal/day). Although dietary assessment was conducted consistently across study periods and no differences in total energy or macronutrient intake were observed between the mango puree and placebo conditions, energy availability was not directly assessed; therefore, the findings cannot be interpreted within the context of relative energy deficiency in sport (RED-S). Third, bioactive compounds in the mango puree were analytically quantified, whereas corresponding analyses were not performed for the placebo, limiting the ability to fully attribute the observed effects to specific bioactive constituents. Finally, the inclusion of only male athletes limits the generalizability of the findings to other populations. Despite these limitations, the randomized, single-blind, crossover design, standardized training routines, and high participant compliance strengthen the internal validity of the study.

## 5. Conclusions

In conclusion, this pilot randomized crossover study demonstrates that mango puree supplementation during regular training was associated with lower adjusted concentrations of inflammatory biomarkers (CRP and IL-6) and creatine kinase, compared with the placebo, in Thai men’s national beach volleyball players. In addition, mango puree supplementation was associated with higher adjusted percentages of CD4+ T cells and a greater CD4/CD8 ratio, while CD8+ T-cell percentage remained unchanged. No significant treatment effects were observed for body composition parameters, blood pressure, or total energy intake. Taken together, these findings indicate that mango puree supplementation was associated with modulation of selected inflammatory, muscle damage, and T-cell-related markers under conditions of intensive training. Given the exploratory design and the limited sample size, the findings should be interpreted within the context of the study population and the selected biomarkers assessed. Further studies in larger and more diverse athletic cohorts, including female athletes and other sports with high training demands, may help to confirm these observations and extend their applicability.

## Figures and Tables

**Table 1:** Bioactive compounds and nutritional composition of the mango puree and placebo used in the intervention.

Variables	Amount (per 200 g Serving)	
	Mango Puree ^a^	Placebo ^b^
Calories (Kcal)	167.9 ± 9.7	166.8
Carbohydrate (g)	39.7 ± 2.5	41.7
Fat (g)	0.4 ± 0.01	0
Moisture (g)	157.7 ± 4.6	158.6
Protein (%N × 6.25) (g)	1.3 ± 0.1	0
Starch (%)	0.7 ± 0.02	ND
Total sugar (g)	33.1 ± 4.1	0
Potassium (mg)	254.0 ± 17.4	<10
Beta-carotene (µg)	746.0 ± 37.2	ND
Total phenolic compounds (mg)	104.7 ± 9.5	ND

Data are presented as the mean ± SD. ^a^ Mango puree data were obtained from laboratory analyses based on three independent batches (*n* = 3). ^b^ Placebo data were calculated using the INMUCAL–Nutrients Version 4.0 database. ND indicates that the parameter was not determined.

**Table 2:** Adjusted general characteristics and body composition parameters following mango puree and placebo supplementation derived from linear mixed-effects models.

Variables	Mango Puree, Adjusted Mean (95% CI)	Placebo, Adjusted Mean (95% CI)	Mean Difference (Mango−Placebo), 95% CI	*p* Value
Body weight (kg)	77.2 (75.9, 78.5)	78.0 (76.8, 79.3)	−0.8 (−2.6, 1.0)	0.347
Waist–hip ratio, WHR	0.71 (0.70, 0.72)	0.70 (0.69, 0.71)	0.01 (−0.01, 0.02)	0.292
Muscle mass (kg)	40.1 (39.3, 40.9)	40.6 (39.8, 41.4)	−0.5 (−1.7, 0.7)	0.391
Fat mass (kg)	6.7 (6.2, 7.2)	7.0 (6.4, 7.5)	-0.2 (−0.9, 0.5)	0.537
Percent fat (%)	8.8 (8.2, 9.4)	8.6 (8.0, 9.2)	0.2 (−0.6, 1.1)	0.573
Total body water (%)	66.8 (65.6, 68.0)	66.6 (65.4, 67.8)	0.2 (−1.5, 1.9)	0.803
Protein (kg)	14.0 (13.8, 14.2)	14.1 (13.9, 14.4)	−0.2 (−0.5, 0.2)	0.323
Mineral (kg)	4.9 (4.8, 5.0)	4.9 (4.8, 5.0)	0.01 (−0.1, 0.9)	0.162
Heart rate (bpm)	69 (67, 72)	66 (64, 68)	3 (0, 7)	0.042 *
Systolic blood pressure, SBP (mmHg)	128 (126, 131)	125 (122, 127)	3 (0, 9)	0.061
Diastolic blood pressure, DBP (mmHg)	76 (73, 80)	72 (68, 75)	4 (−1, 10)	0.101

Data are presented as adjusted means and 95% confidence intervals (CI) derived from linear mixed-effects models. Mean differences represent mango puree minus placebo. *p* values refer to the fixed effect of treatment. * indicates *p* < 0.05.

**Table 3:** Distribution of energy-yielding macronutrients during the baseline, mango puree, and placebo study periods.

Macronutrients	Baseline	Mango Puree ^ns1^	Placebo ^ns1, ns2^
Carbohydrate (%)	42.9 ± 12.8	46.1 ± 11.9	39.7 ± 13.6
Fat (%)	31.1 ± 10.9	24.8 ± 5.2	37.4 ± 14.7
Protein (%)	26.0 ± 10.3	29.1 ± 12.8	22.9 ± 7.9

Data are presented as mean ± SD (*n* = 15). ^ns1^ indicates no significant difference among the baseline, mango puree, and placebo study periods. ^ns2^ indicates no significant difference between the mango puree and placebo study periods.

**Table 4:** Energy and macronutrient intake during the baseline, mango puree, and placebo study periods.

Energy and Nutrients	Baseline	Mango Puree ^ns1^	Placebo ^ns1, ns2^
Energy (Kcal)	2066 ± 609	2074 ± 630	2058 ± 588
Carbohydrate (g)	221.6 ± 65.4	239.2 ± 72.6	204.1 ± 58.4
Fat (g)	71.5 ± 21.0	57.1 ± 17.3	85.5 ± 24.4
Protein (g)	134.3 ± 39.7	151.2 ± 46.0	118.0 ± 33.7

Data are presented as mean ± SD (*n* = 15). ^ns1^ indicates no significant difference among the baseline, mango puree, and placebo study periods. ^ns2^ indicates no significant difference between the mango puree and placebo study periods.

## Data Availability

The data presented in this study are available on request from the corresponding author due to confidentiality concerns, as the participants are national team athletes and individual-level data cannot be publicly disclosed.

## Abbreviations

The following abbreviations are used in this manuscript:

CRP	C-reactive protein
DBP	Diastolic blood pressure
ROS	Reactive oxygen species
SBP	Systolic blood pressure
CK	Creatine kinase
IL-6	Interleukin-6
